# 

**Published:** 1843-09-30

**Authors:** 


☞ Published every alternate Saturday, and subscriptions received, by J. C. TURNPENNY, N. E. corner of Spruce and Tenth streets. Price, Three Dollars a year, invariably in advance. Two copies sent to the same address, Five Dollars.

Communications and Letters to be addressed (always pre-paid) to the Editor of the Medical Examiner, Philadelphia.

Postmaster's franks can always be obtained for remittances.

This journal is subject to newspaper postage only.

MERRIHEW AND THOMPSON, PRINTERS, 7 CARTER'S ALLEY.

